# Supplementary material for: Investigating the correlation between candidate teachers’ acceptance of generative artificial intelligence and artificial intelligence literacy across various disciplines
Source: PLoS One. 2026 Mar 5;21(3):e0342853. doi: 10.1371/journal.pone.0342853 (PMC12962539; doi:10.1371/journal.pone.0342853)
Supplement: Attachment — Submitted filename: Response to Reviewers.docx [file pone.0342853.s002.docx]

Response to Reviewer 1

| The manuscript inconsistently refers to “General Artificial Intelligence (GAI)” instead of “Generative AI.” This is potentially misleading, as “general AI” has a different meaning in the literature. The terminology should be standardized throughout the paper. | Thank you for pointing out this important issue. We agree that the term “General Artificial Intelligence (GAI)” may be misleading. The manuscript has been revised to consistently use “Generative Artificial Intelligence (GenAI)” throughout the text. All instances of “GAI” and “general AI” have been corrected, and the terminology has been standardized accordingly. |
| --- | --- |
| The authors should explicitly define “AI literacy” and situate it within established frameworks (e.g., Long & Magerko, 2020; UNESCO, 2021). | We have revised the manuscript to explicitly define AI literacy and to situate the concept within established frameworks. Specifically, we incorporated the definitions and competency-based framework proposed by Long and Magerko (2020) and aligned our conceptualization with UNESCO’s (2021) human-centered and ethical perspective on AI literacy. These revisions have been added to the section. |
| The exclusive focus on pre-service teachers is a limitation. While their perspectives are valuable, they are not yet fully immersed in the realities of classroom practice. The authors should acknowledge this limitation and discuss how findings may differ from those of in-service teachers. | We have revised the manuscript to explicitly acknowledge the exclusive focus on pre-service teachers as a limitation of the study in the Limitations section. In the Discussion, we further elaborated on how pre-service teachers’ beliefs and intentions toward AI are shaped during initial teacher education, while in-service teachers’ perceptions may be more strongly influenced by contextual and institutional factors such as curriculum demands, classroom management responsibilities, and school-level policies. Accordingly, we clarified that the findings primarily reflect emerging perceptions and intentions rather than established classroom practices and cautioned against overgeneralization. We also suggested that future research include in-service teachers to provide a more comprehensive and practice-oriented perspective. |
| To strengthen the discussion, the authors should integrate literature on faculty and in-service teacher adoption of generative AI, which provides a more grounded perspective. Relevant studies include: ◦ Miranda-González, F. J., & Chamorro-Mera, A. (2025). Exploring the adoption of generative artificial intelligence tools among university teachers. Higher Education Research & Development. https://doi.org/10.1080/07294360.2025.2559648 ◦ Chamorro-Mera, A., & Miranda-González, F. J. (2025). Gen-AI tools in academia: A cluster analysis of university faculty adoption. Multidisciplinary Journal for Education, Social and Technological Sciences, 12(2). https://doi.org/10.4995/muse.2025.23908 ◦ Almisad, B., & Aleidan, A. (2025). Faculty perspectives on generative artificial intelligence: Insights into awareness, benefits, concerns, and uses. Frontiers in Education, 10, Article 1632742. https://doi.org/10.3389/feduc.2025.1632742 ◦ Singh, S., & Strzelecki, A. (2025). Academics as adopters of generative AI: An application of diffusion of innovations theory. Education and Information Technologies, 30(11), 20495–20522. https://doi.org/10.1007/s10639-025-13835-8 | We have revised the manuscript to acknowledge the limitation of focusing on pre-service teachers and discuss how their perceptions may differ from in-service teachers. We have enhanced the Discussion section by incorporating recent literature on generative AI adoption among in-service teachers and faculty. We integrated findings from studies showing how experienced educators' engagement with generative AI relates to institutional contexts, ethical concerns, and professional practice (Miranda-González & Chamorro-Mera, 2025; Chamorro-Mera & Miranda-González, 2025; Almisad & Aleidan, 2025; Singh & Strzelecki, 2025). This integration contrasts pre-service teachers' perceptions with in-service educators' practice-based perspectives and clarifies both groups' contributions. These revisions strengthen the discussion and enhance the findings' interpretability. |
| The description of the mixed-methods design is clear, but the rationale for choosing pre-service teachers over in-service teachers should be elaborated. | We elucidated that pre-service teachers are at a formative stage in developing beliefs, attitudes, and intentions regarding emerging educational technologies, including generative AI. This cohort was chosen to capture initial perceptions before the classroom and institutional constraints typically encountered by in-service teachers. We contend that this clarification enhances coherence between the research design, participant selection, and study objectives. |
| The qualitative sample (48 participants) is relatively small compared to the quantitative sample. The authors should justify how saturation was achieved and how representativeness was ensured. | We have revised the manuscript to further justify the qualitative sample size and to clarify how saturation and representativeness were addressed. |
| The data availability statement is inconsistent: one section claims full availability, while another restricts access due to personal data. This contradiction must be resolved. | The quantitative data supporting the findings of this study have been uploaded to the system (figshare) and shared with the editors. Due to ethical reasons and the inclusion of personal data (participants were assured that audio recordings would not be shared with anyone other than the researchers), the qualitative interview data are not publicly available. |
| The findings are presented clearly, but the discussion could be more strongly tied to theoretical frameworks such as the Technology Acceptance Model (TAM) or Diffusion of Innovations Theory. | We have strengthened the Discussion section by more explicitly linking the findings to established theoretical frameworks, particularly the Technology Acceptance Model (TAM) and Diffusion of Innovations Theory |
| The authors should expand on the implications of the very low percentage of participants (6.1%) who received formal AI training. This is a critical finding that deserves more emphasis in terms of curriculum design and policy recommendations. | We expanded the Discussion and Implications sections to highlight the low proportion of participants (6.1%) receiving formal AI training. We elaborated on how this reveals a critical gap in teacher education and its implications for curriculum design, including embedding AI literacy in core coursework. We added policy implications, emphasizing institutional and national guidelines for systematic AI training for future teachers. These additions strengthen the study's contribution to educational practice and policy. |
| The abstract and introduction are overly verbose. Shorter, more concise sentences would improve readability. | We have revised the abstract and introduction to improve clarity and readability by shortening sentences and reducing unnecessary verbosity. |
| The discussion should include a stronger reflection on ethical concerns (bias, privacy, transparency) and how these intersect with teacher education. | We have strengthened the Discussion section by explicitly expanding our reflection on ethical concerns related to generative AI, including bias, data privacy, and transparency. |

Response to Reviewer 2

| The manuscript would benefit from citing two recent studies directly aligned with generative AI awareness and the pedagogical use of AI in education. I strongly recommend adding the following references to strengthen the theoretical framework and discussion:  Semerci Şahin et al. (2025) – development of the Generative AI Awareness Scale for secondary school students, which directly relates to AI literacy and awareness measurement approaches. Reference: Semerci Şahin, R., Özbay, Ö., Çınar Özbay, S., & Durmuş Sarıkahya, S. (2025). Development of the generative artificial intelligence awareness scale for secondary school students in Türkiye. European Journal of Pediatrics, 184(9), 585. https://doi.org/10.1007/s00431-025-06435-8  Sarıkahya et al. (2025) – a qualitative study examining faculty members' experiences with ChatGPT in nursing education, highly relevant for understanding generative AI acceptance, pedagogical challenges, and literacy gaps. Reference: Sarıkahya, S. D., Özbay, Ö., Torpuş, K., Usta, G., & Özbay, S. Ç. (2025). The impact of ChatGPT on nursing education: A qualitative study based on the experiences of faculty members. Nurse Education Today, 152, 106755. https://doi.org/10.1016/j.nedt.2025.106755  These studies will specifically enrich the authors’ sections on GAI acceptance, literacy, and discipline-based differences in attitudes toward AI. | We have revised the manuscript to incorporate both suggested studies, which are highly aligned with our focus on generative AI acceptance, literacy, and pedagogical use. |
| --- | --- |
| Introduction – Reduce Repetition Some sentences repeat similar ideas about AI's growing role in education. A brief refinement would improve clarity | We have revised the abstract and introduction to improve clarity and readability by shortening sentences and reducing unnecessary verbosity. |
| Conceptual Clarification The manuscript alternates between “GAI” and “General Artificial Intelligence.” Please ensure consistent terminology throughout. | Thank you for pointing out this important issue. We agree that the term “General Artificial Intelligence (GAI)” may be misleading. The manuscript has been revised to consistently use “Generative Artificial Intelligence (GenAI)” throughout the text. All instances of “GAI” and “general AI” have been corrected, and the terminology has been standardized accordingly. |
| Figures 4 and 5 The thematic maps are informative but somewhat difficult to read due to resolution. Increasing clarity or resolution would strengthen presentation | We have revised Figures 4 and 5 to improve clarity and readability by increasing their resolution and adjusting the layout and font sizes |
| Discussion Section The discussion is thorough; however, condensing overlapping ideas and explicitly linking the results to the newly suggested studies would improve coherence. With these minor revisions addressed particularly the inclusion of the two recent and relevant references the manuscript will be suitable for publication. | We have revised the Discussion section by condensing overlapping ideas to improve coherence and readability. In addition, we explicitly integrated and discussed the two newly suggested studies (Semerci Şahin et al., 2025; Sarıkahya et al., 2025), directly linking our findings to their contributions on generative AI awareness, literacy, and pedagogical use. |
