# Supplementary material for: Investigating the correlation between candidate teachers’ acceptance of generative artificial intelligence and artificial intelligence literacy across various disciplines
Source: PLoS One. 2026 Mar 5;21(3):e0342853. doi: 10.1371/journal.pone.0342853 (PMC12962539; doi:10.1371/journal.pone.0342853)
Supplement: Attachment — Submitted filename: Response to Reviewers and Editor_2nd.docx [file pone.0342853.s003.docx]

We thank Reviewer 1 for his/her comment: “I appreciate the revisions made to the manuscript. The authors have adequately addressed all the comments raised in the previous review round, and the changes introduced have improved the clarity and overall quality of the paper. I do not identify any remaining issues that require further modification. In light of this, I consider the manuscript suitable for publication and recommend its acceptance.” There have been no corrections from the reviewers regarding the second revision. As the authors, we have revised our manuscript in light of the “additional editor comments” and uploaded the 'Revised Manuscript with Track Changes' file. In addition, we would like to mention that in our final review of the article, we provided the numerical values in the table using both dots and commas, and that it was subsequently revised to use dots in order to establish a common format for the numerical values. Best regards.

**Additional Editor Comments and Responses**

**(i)** Revise the Data Availability Statement to provide a clear, non-discretionary procedure for accessing the underlying dataset (and clarify whether a de-identified dataset can be shared

**R (i):** In order to preserve author anonymity during the review process, the initial version of the manuscript referred only to the platform where the data were deposited. Following this comment, the Data Availability Statement has been revised, and the DOI corresponding to the dataset has now been explicitly included in the manuscript to ensure transparent and non-discretionary access to the underlying data.

**(ii)** clarify the wording and measurement units for GenAI usage categories (as the current distribution may be open to misinterpretation

**R (ii):** We appreciate your comment. We wish to clarify that the usage of GenAI is operationalized and reported in Table 1 as Daily GenAI Usage (hours/day), which explicitly denotes the measurement unit employed for categorizing participants' GenAI use. We believe this phrasing minimizes potential ambiguity in interpreting the distribution. We understand from your comment that the primary concern pertains to the clarity of wording and measurement units; however, we are willing to make further revisions should you deem additional clarification or modifications necessary.

**(iii)** complement the main statistical comparisons with appropriate effect sizes (and, where feasible, confidence intervals);

**R (iii):** The desired statistical results have been added to the article.

**(iv)** slightly improve the integration of quantitative and qualitative findings by briefly stating how survey results informed the interview guide and by linking selected quotes to key quantitative patterns.

**R (iv):** “The results indicated that participants' levels of acceptance of GenAI and AIL varied according to their departmental affiliation. To explore the potential reasons for these departmental differences in greater depth, qualitative interview instruments were developed.” added the article.
